# *Coleus forskohlii* Extract Supplementation in Conjunction with a Hypocaloric Diet Reduces the Risk Factors of Metabolic Syndrome in Overweight and Obese Subjects: A Randomized Controlled Trial

**DOI:** 10.3390/nu7115483

**Published:** 2015-11-17

**Authors:** Hayley L. Loftus, Katie J. Astell, Michael L. Mathai, Xiao Q. Su

**Affiliations:** Centre for Chronic Disease, College of Health and Biomedicine, Victoria University, P.O. BOX 14428 MCMC, Melbourne 8001, Australia; hayley.loftus@live.vu.edu.au (H.L.L.); katie.astell@live.vu.edu.au (K.J.A.); michael.mathai@vu.edu.au (M.L.M.)

**Keywords:** *Coleus forskohlii*, appetite, waist circumference, hip circumference, metabolic parameters

## Abstract

Limited studies have shown that *Coleus forskohlii* extract may aid in weight management. This randomized, double blind placebo-controlled clinical study assessed the effects of supplementation with *C. forskohlii* extract on key markers of obesity and metabolic parameters in overweight and obese individuals. Thirty participants completed the trial and they were randomly assigned to receive either 250 mg of *C. forskohlii* extract (*n* = 15) or a placebo twice daily for 12 weeks. All participants were advised to follow a hypocaloric diet throughout the study. Body weight, body mass index (BMI), waist and hip circumference, and waist to hip ratio, were monitored fortnightly. Dietary intake was assessed at the baseline and weeks 4, 8 and 12. Appetite was assessed using visual analogue scales and blood samples were analyzed for plasma lipids, ghrelin, leptin, glucose and insulin at the baseline and end of the intervention. Significant reductions to waist and hip circumference (*p* = 0.02*; p* = 0.01, respectively) were recorded in both experimental and placebo groups after the 12 week intervention. Furthermore, high density lipoprotein-cholesterol (HDL-C) was significantly increased (*p* = 0.01) in both groups. The experimental group showed a favorable improvement in insulin concentration and insulin resistance (*p* = 0.001; 0.01 respectively) compared to the placebo group. These findings suggest that *C. forskohlii* extract in conjunction with a hypocaloric diet may be useful in the management of metabolic risk factors.

## 1. Introduction

Metabolic syndrome is a common cardio-metabolic disorder that is characterized by the simultaneous occurrence of cardiovascular risk factors including central obesity, dyslipidemia, hyperglycemia and hypertension [1]. Central obesity is a major predisposing factor to the development of metabolic syndrome and is a key component of metabolic syndrome [2,3,4]. The exact mechanism by which metabolic syndrome develops is not yet fully understood but it appears to be related to insulin resistance and excessive free fatty acid release from intra-abdominal adipocytes [5,6,7]. It is also believed that a pro-inflammatory and pro-thrombotic state contribute to the pathogenesis of the syndrome [8]. Metabolic syndrome poses a major health risk for the development of cardiovascular disease (CVD) and type II diabetes mellitus (T2DM), thus an effective therapeutic approach is in high demand for those who are at risk [9]. The majority of individuals affected with metabolic syndrome are overweight or obese and therefore treatment is primarily focused on weight reduction [10,11,12].

Lifestyle interventions such as dietary modification and physical activity remain the cornerstone of weight loss treatment. In theory, reducing energy intake and increasing energy expenditure will elicit weight loss [10]. However, obesity is a multifactorial condition encompassing complex environmental and genetic influences leading to overconsumption and reduced physical activity making weight loss a challenging issue [13]. As the prevalence of overweight and obesity continues to rise at an alarming rate, more effective weight-control therapies are required [14]. The availability and popularity of natural weight loss supplements has increased drastically over recent years. *Coleus forskohlii* auct. (*Plectranthus barbatus* Andrews*)* extract is among those natural products that shows promising therapeutic anti-obesity potential [15].

**Figure 1:**
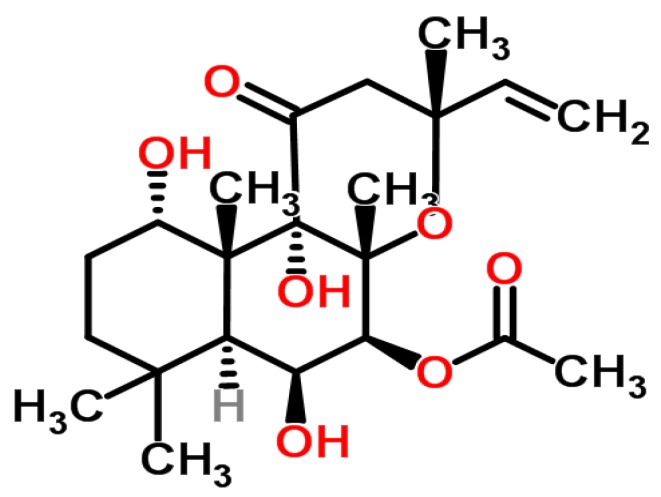
Chemical structure of forskolin.

*Coleus forskohlii* auct. is a perennial plant of the Lamiaceae (mint) family and is native to Nepal, Thailand and India. Forskolin is the major active constituent of *C. forskohlii* which is of clinical interest [16]. Forskolin (7β-Acetoxy-8, 13-epoxy-1α, 6β, 9 α-trihydroxy-labd-14-ene-11-one) (Figure 1) is a labdane diterpene that was first isolated from the plant in 1974. Forskolin is extracted from the tuberous roots of the plant and thus far*, C. forskohlii* is the only species known to contain significant amounts of the bioactive component [17]. It has been documented that forskolin increases the rate of lipolysis via cyclic adenosine monophosphate (cAMP) accumulation by mechanisms independent of hormonal stimulation both *in vitro* [18,19] and in animal models [20,21]. Furthermore, forskolin also directly activates hormone sensitive lipase by phosphorylation of protein kinase A resulting in further lipolysis and release of free fatty acids [19].

Limited human studies have indicated that supplementation with *C. forskohlii* extract elicits favorable changes to body composition. Godard*, et al.* [22] found a significant reduction of body fat percentage in overweight and obese men after 12 weeks of supplementation with *C. forskohlii* extract. Similar results have also been documented in preliminary open-label trials conducted on overweight and obese female volunteers [23] and men and women within the healthy BMI range (24.92 ± 0.87) [24]. In addition, Henderson*, et al.* [25] reported significant reductions in dietary intake in overweight women but did not find any significant differences in subjective appetite sensations or body composition after 12-weeks of supplementation. The aim of the present study was to evaluate the effects of 12-weeks supplementation with *C. forskohlii* extract on dietary intake and appetite, anthropometric measures, and metabolic parameters in overweight and obese individuals. To the best of our knowledge, this is the first study investigating the efficacy of *C. forskohlii* extract on the appetite hormones, leptin and ghrelin, and insulin sensitivity in overweight and obese individuals.

## 2. Experimental Section

### 2.1. Participants and Randomization

**Figure 2:**
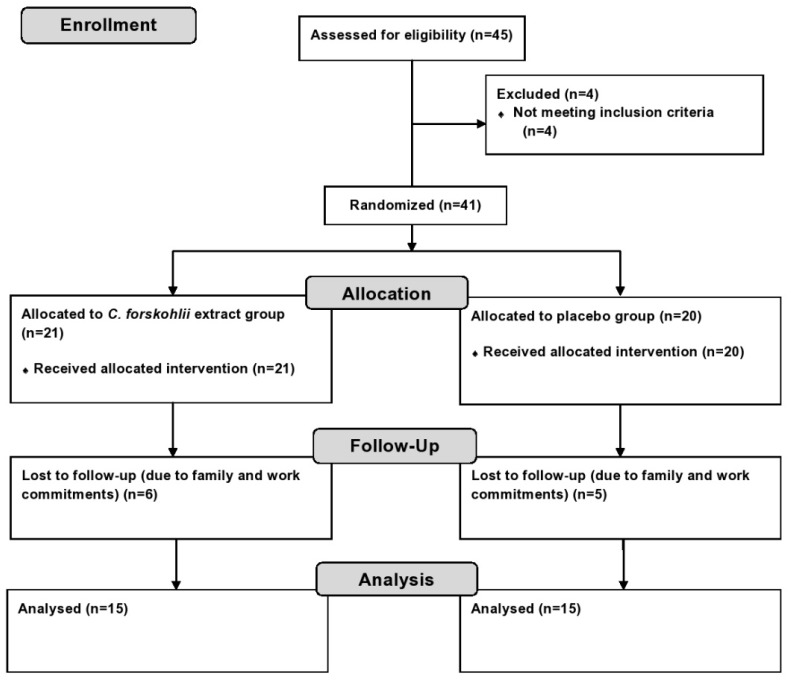
Flow chart of study design.

This study was a double blind, placebo-controlled randomized clinical trial conducted at Victoria University, Melbourne, Australia. Participants were recruited through the general community, staff members and students from May to July 2014. Forty-one overweight or obese participants (12 males and 29 females) were enrolled in the trial (Figure 2). All participants were screened to assess eligibility prior to enrolling and informed written consent was obtained. The eligibility criteria included: BMI > 25 m² or waist circumference > 94 cm (male) and > 80 cm (female), 20–65 years, non-smoker, non-pregnant or lactating, non-type 1 diabetic, no present liver, kidney or heart disease, not susceptible to peptic ulcers and not taking any medication for weight loss, hypertension or thrombocytosis. Prior to randomization, an initial assessment was conducted to measure participant’s weight, height, waist and hip circumference, blood pressure (BP) and heart rate (HR). BMI and waist-to-hip ratio (WHR) were then calculated. This information was used to randomly allocate participants into either the treatment or the control group by the chief investigator. Stratification method of randomization was carried out to ensure that the baseline characteristics were evenly distributed amongst groups to avoid potential confounding results. The method used to ensure allocation concealment was sequentially numbered containers. The containers were equal in weight, similar in appearance and tamper-proof. The principal investigator implemented the allocation sequence and assigned the participants into their groups. The capsules were opaque and indistinguishable in appearance, size, texture and smell. The taste of the capsules was identical provided that they were swallowed whole as instructed. Staff and participants involved in the intervention process of the trial were blinded to group assignment. The randomization code was broken only after data analysis was completed. The study was approved by the Human Ethics Research Committee of Victoria University (VUHERC approval number 14-020). Trial registration: Australia New Zealand Clinical Trial Registry (ANZCTR): ACTRN12614000305628.

### 2.2. Experimental Design

Olive Life Sciences, India, manufactured and supplied the *C. forskohlii* extract and the placebo capsules for this study. The *C. forskohlii* extract was obtained from the sun-dried tuberous roots of the plant by ethanol grade extraction. The extract was standardized to contain 10% forskolin. Once extracted, the *C. forskohlii* was then blended with maltodextrin and aerosil, and then pulverized and packaged into capsules containing 250 mg of *C. forskohlii* extract. The dosage was determined based on the previous study by Kamohara and Noparatanawong [24]. Placebo capsules contained 250 mg of maltodextrin. Participants were instructed to take one capsule twice daily 30 min before main meals for 12-weeks. Compliance to adhering to this regimen was monitored by providing participants with a fortnightly capsule calendar to mark of their daily doses and also by counting any remaining capsules (if any) from their fortnightly supply.

At the beginning of the study (week 1) anthropometric parameters (weight, height, BMI, waist and hip circumference and WHR), BP, HR and fasting plasma biochemical analysis including ghrelin, leptin, insulin, triglycerides (TG), total cholesterol (TC), high density lipoprotein cholesterol (HDL-C), low density lipoprotein cholesterol (LDL-C) and glucose were performed.

In addition, an appetite assessment, food frequency questionnaire (FFQ) [26] and physical activity questionnaire [27] were also conducted. These investigations were undertaken again at the conclusion of the study (week 12). Furthermore, dietary intake and physical activity were controlled during the intervention. Both groups received consistent dietary advice once per fortnight and were advised to maintain their routine physical activity levels. Dietary intake was monitored through 3-day food diaries obtained at the baseline and weeks 4, 8 and 12 of the study and participation in physical activity was monitored fortnightly. Participants in both groups were given standard dietary advice and instructed to follow a hypocaloric diet (deficit of approximately 500 kcal/day of estimated energy requirements) based on the Adult Weight Management Evidence-Based Nutrition Practice Guidelines [28]. All anthropometric measurements were taken during fortnightly appointments. At these visits, participants were also asked to report if any negative side effects were experienced.

### 2.3. Anthropometry and Physiological Measurements

All anthropometric (weight, height, waist and hip circumference) and physiological measurements (BP and HR) were performed using standard technique and equipment [29]. BMI was calculated using the following formula: BMI (kg/m^2^) = weight/height^2^. WHR was calculated using the following formula: WHR = waist circumference (cm)/hip circumference (cm). BP and HR were measured after the participant had been seated and relaxed for at least five minutes using an automated digital BP monitor (Omron Healthcare, Kyoto, Japan). All measurements were taken in duplicate and the mean of the measurements was used as the final result.

### 2.4. Biochemical Analyses of Metabolic Parameters

At the baseline and at the completion of the 12-week trial, fasting blood samples were collected for the analysis of plasma leptin, ghrelin, insulin and lipids (TC, HDL-C and TG). Participants were asked to refrain from exercise and fast for approximately 10 hours (overnight) prior to the blood collection. Approximately 10 mL of venous blood was drawn from the cubital vein using a winged needle and BD ethylene diamine tetraacetic acid (EDTA) vacutainer. Immediately after collection, blood samples were centrifuged for 10 min at 3000 *g* and 4 °C. The plasma was aliquoted into cryotubes and was stored at −80 °C until analysis. A digital glucometer (Abott Diabetes Care Inc., Melbourne, Australia) was used to determine fasting blood glucose concentration.

Plasma concentrations of TG, TC and HDL-C were determined using commercial colorimetric assay kits (Thermo Fisher Scientific Inc., Middletown, OH, USA) and analyzed with an automated spectrophotometer in accordance with the manufacturer’s instructions. Plasma LDL-C concentration was derived using the Friedewald equation [30]: LDL-C = TC − HDL-C − (TG/2.7) (mmol/L).

The concentration of plasma leptin and insulin was determined with commercial (Mercodia Inc. Uppsala, Sweden; Abcam Inc., Cambridge, MA, USA) enzyme linked immunosorbent assay (ELISA) kits in accordance to the manufacturer’s instructions using an automated spectrophotometer. Total plasma ghrelin concentration was measured by radioimmunoassay [31] using a commercial kit (Linco Research, Saint Charles, MI, USA) in accordance to the manufacturer’s instructions. Insulin resistance was determined using the homeostatic model assessment of insulin resistance (HOMA-IR) [32] with the following equation: HOMA-IR = fasting insulin (µU/L) × fasting glucose (nmol/L)/22.5.

### 2.5. Appetite Assessment

Fasting and postprandial appetite was assessed at the baseline and at the end of the intervention using a previously validated visual analogue scale (VAS) method [33]. Briefly, participants underwent the VAS following an overnight fast of 10 hours and then the VAS was repeated again after the ingestion of a standardized buffet-style test breakfast. Appetite sensations were recorded in the VAS by participants placing a vertical stroke along a 100 mm horizontal line to represent: how hungry they feel (not hungry-hungry); how full they feel (not full-full); how nauseous they feel (not nauseous-nauseous); how drowsy they feel (not drowsy-drowsy); how anxious they feel (not anxious-anxious); how strong their desire to eat is (very weak-very strong); how much food they think they could eat (none-a large amount). Participants were given thirty minutes to consume their test breakfast meal. After obtaining the energy content of the test meal, the satiety quotient (SQ) was then calculated for each appetite sensation (AS) and all time point SQs were averaged (0–180 min) to evaluate the satiating efficiency of the meal [34] using the following equation: SQ (mm/ kcal) = (fasting AS − post meal AS/energy content of test meal) × 100.

### 2.6. Dietary Intake

Participants recorded all food and fluid intake for three days (two weekdays and one weekend day) at baseline and weeks 4, 8 and 12 on dietary recall forms. The dietary intake of energy and nutrients was then quantified by the researcher using Food Works Professional (Xyris software Pty Ltd., Highgate Hill, Australia). Additionally, at the baseline and week 12 participants completed a previously validated [35,36] FFQ (Dietary Questionnaire for Epidemiological Studies (DQES) version 2, Cancer Council, Melbourne, Australia) that assessed their dietary history for energy, macronutrient and micronutrient intake during the past three months.

### 2.7. Statistical Analysis

The sample size for the trial (a minimum of 13 in each group) was determined by statistical power analysis (two tailed *t*-test at the 0.05 significance level) based on the previous findings of Henderson, *et al.* [25] for the power of 90% of expected differences in total energy intake-one of the major measured outcomes of the trial. All data are presented as mean ± standard deviation unless specified otherwise and were analyzed using SPSS, v19 (IBM, Chicago, IL, USA). Independent *t*-tests were performed to compare all baseline data between groups. Mixed model analysis of variance (ANOVA) was used to analyze the effects of the intervention, time, and the interaction between the intervention and time with pairwise comparisons (adjusted for multiple comparisons by Bonferroni’s *post-hoc* test). Results were considered statistically significant when *p* < 0.05.

## 3. Results

Out of 41 enrolled participants 30 completed the trial (control group: *n* = 15, 12 females and 3 males, experimental group: *n* = 15, 12 females and 3 males). Eleven participants were lost to follow up due to time constraints with work and family commitments (Figure 2). There were no significant differences observed between the two groups at the baseline of the intervention for age, weight, BMI, waist circumference, hip circumference or WHR (Table 1).

**Table 1:** Baseline physical characteristics of the participants at randomization.

Parameter	Experimental Group (*n* = 15)	Placebo Group (*n* = 15)	*p* Value
Age (years)	40.9 ± 13.8	41.8 ± 14.2	0.9
Body weight (kg)	90.9 ± 13.9	93.4 ± 21.2	0.9
Body mass index (kg/m²)	31.9 ± 4.0	33 ± 6.4	0.7
Waist circumference (cm)	105.4 ± 11.1	108.5 ± 17.8	0.8
Hip circumference (cm)	112.7 ± 8.2	113.2 ± 12.0	0.8
Waist to hip ratio	0.93 ± 0.08	0.96 ± 0.1	0.7

Values are expressed as mean ± standard deviation (SD), *n* = number of subjects.

### 3.1. Anthropometric and Physiological Parameters

Table 2 shows anthropometric measurements and physiological parameters taken at the baseline, midway and at the end of the intervention. A significant time effect (*p* < 0.05) was observed in waist and hip circumference between week 12 and the baseline with mean reductions of 4.5 ± 1.7 cm and 3.5 ± 1.1 cm recorded in waist circumference in the experimental and placebo groups, respectively; and 3.4 ± 1.1 cm and 3.0 ± 1.2 cm reductions in hip circumference in the experimental and placebo groups, respectively (Table 2). There were no marked differences observed in other anthropometric or physiological parameters (Table 2).

**Table 2:** Anthropometric and physiological parameters of participants at baseline, week 6 and week 12 of intervention with C. *forskohlii* extract (*n* = 15 in each group).

Parameter	Study Group	Baseline	Week 6	Week 12	*p* Value	
Weight	Experimental	89.5 ± 13.2	88.6 ± 12	88 ± 11.4	Group	0.3
(kg)	Placebo	92.9 ± 21.2	92.2 ± 20.5	91.5 ± 21	Time	0.3
					Interaction	1
BMI	Experimental	31.8 ± 3.9	30.7 ± 3.6	30.4 ± 3.4	Group	0.7
(kg/m^2^)	Placebo	32.8 ± 6.5	32.6 ± 6.4	32.2 ± 6.2	Time	0.1
					Interaction	1
Waist	Experimental	106.9 ± 11.7	103.6 ± 9.8	102.4 ± 11.3	Group	1.0
(cm)	Placebo	106.1 ± 11.2	103.7 ± 11.6	102.6 ± 15 *	Time	0.02
					Interaction	0.7
Hip	Experimental	112.3 ± 5.9	109.8 ± 5	108.9 ± 5.4	Group	0.3
(cm)	Placebo	115.3 ± 13.2	113.8 ± 11.9	112.3 ± 12.6 *	Time	0.01
					Interaction	0.6
WHR	Experimental	0.95 ± 0.09	0.94 ± 0.08	0.94 ± 0.08	Group	0.3
	Placebo	0.92 ± 0.08	0.91 ± 0.09	0.91 ± 0.08	Time	0.3
					Interaction	0.8
Systolic BP	Experimental	125 ± 17	128 ± 13	125 ± 13	Group	0.3
(mmHg)	Placebo	128 ± 13	132 ± 12	130 ± 10	Time	0.4
					Interaction	0.8
Diastolic BP	Experimental	79 ± 8	79 ± 9	77 ± 9	Group	0.6
(mmHg)	Placebo	83 ± 8	80 ± 8	78 ± 10	Time	0.1
					Interaction	0.9
Heart Rate	Experimental	74 ± 13	75 ± 12	73 ± 13	Group	0.3
(bpm)	Placebo	76 ± 14	73 ± 11	74 ± 12	Time	0.1
					Interaction	0.6

Values are expressed as mean ± SD. * *p* < 0.05, compared to baseline for each intervention. WHR, waist to hip ratio; BP, blood pressure.

### 3.2. Metabolic Parameters

**Figure 3:**
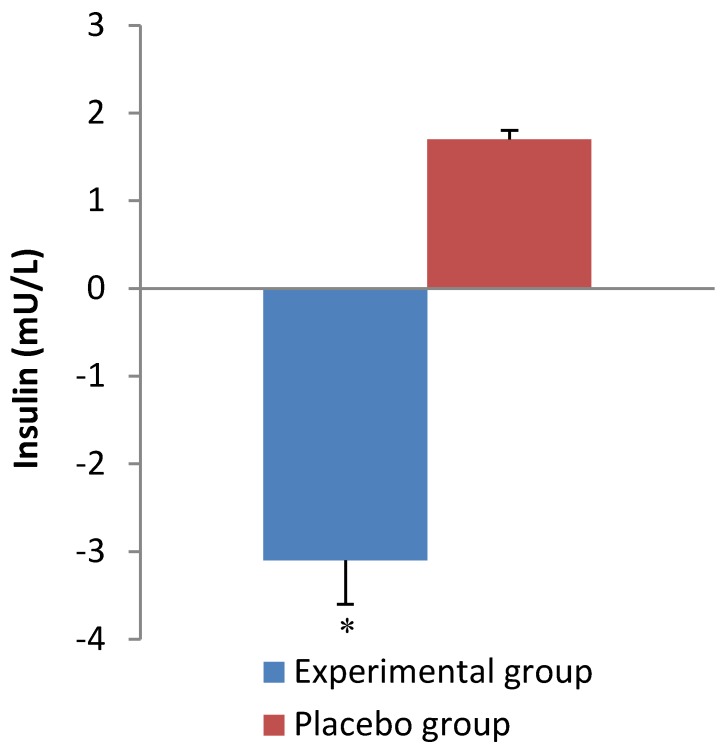
Change in insulin over the intervention period. Values are expressed as mean ± SEM. * *p* < 0.05, compared with placebo group.

**Figure 4:**
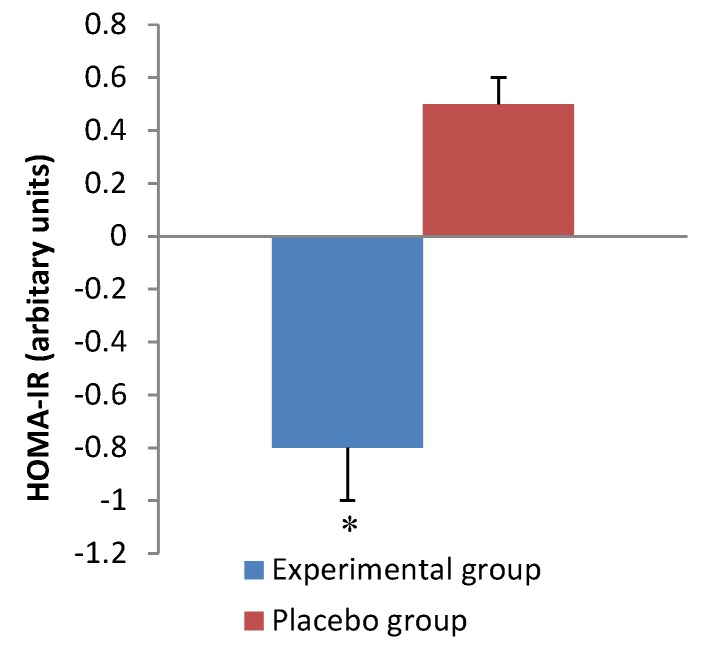
Change in insulin resistance over the intervention period. Values are expressed as mean ± SEM. * *p* < 0.05, compared with placebo group.

Table 3 shows the results of metabolic parameters at the baseline and end of the intervention. Supplementation with *C. forskohlii* extract for 12 weeks significantly reduced (*p =* 0.001) insulin concentration compared with the control group (Figure 3). Consistently, there was a significant difference observed (*p* = 0.01) in HOMA-IR between the experimental and control group after the 12-week intervention (Figure 4). HDL-C was significantly increased (*p* = 0.01) at week 12 compared to baseline in both groups. No significant differences were observed for blood glucose, TG, TC, HDL to LDL-C ratio, ghrelin or leptin in either group after the 12-week intervention.

**Table 3:** Metabolic parameters of participants at baseline and week 12 of intervention with *C*. *forskohlii* extract (*n* = 15 in each group).

Parameter	Baseline	Week 12	*p* Value	
*Fasting blood glucose (mmol/L)*			Group	0.9
Experimental	5.3 ± 0.5	5.5 ± 1.0	Time	0.8
Placebo	5.3 ± 1.0	5.5 ± 1.4	Interaction	1
*Triglycerides (mmol/L)*			Group	0.2
Experimental	1.2 ± 0.5	1.1 ± 0.7	Time	0.5
Placebo	1.5 ± 1.0	1.4 ± 0.6	Interaction	0.8
*Total cholesterol (mmol/L)*			Group	0.9
Experimental	4.1 ± 1.2	3.7 ± 0.9	Time	0.08
Placebo	4.1 ± 1.1	4.5 ± 0.7	Interaction	0.9
*HDL-C (mmol/L)*			Group	0.4
Experimental	0.9 ± 0.4	1.2 ± 0.5 *	Time	0.01
Placebo	1.1 ± 0.6	1.3 ± 0.5 *	Interaction	0.7
*LDL-C (mmol/L)*			Group	0.6
Experimental	2.76 ± 1.0	2.83 ± 1.0	Time	0.5
Placebo	2.5 ± 1.7	2.8 ± 0.9	Interaction	0.7
*HDL:LDL (mmol/L)*			Group	0.6
Experimental	0.4 ± 0.2	0.5 ± 0.3	Time	0.7
Placebo	0.5 ± 0.1	0.6 ± 0.4	Interaction	0.9
*Insulin (mU/L)*			Group	0.8
Experimental	9.6 ± 5.7	6.1 ± 2.3	Time	0.2
Placebo	6.8 ± 3.8	8.5 ± 4.1	Interaction	0.001
*HOMA-IR*			Group	1
Experimental	2.3 ± 1.5	1.5 ± 0.7	Time	0.5
Placebo	1.4 ± 0.9	2.1 ± 1.4	Interaction	0.01
*Ghrelin (pg/mL)*			Group	0.6
Experimental	622 ± 127	647 ± 143	Time	0.4
Placebo	643 ± 209	686 ± 208	Interaction	0.9
*Leptin (pg/mL)*			Group	1
Experimental	33938 ± 4117	38783 ± 3451	Time	0.8
Placebo	36286 ± 5120	32701 ± 9609	Interaction	0.5

Values are expressed as mean ± SD. * *p* < 0.05, compared to baseline for each intervention. HDL-C, high-density lipoprotein cholesterol; LDL-C, low-density lipoprotein cholesterol; HOMA-IR, Homeostatic model assessment of insulin resistance

### 3.3. Dietary Intake and Physical Activity

Both food diaries and FFQ were used to assess dietary intake. The results from the food diary analyses at the baseline and weeks 4, 8 and 12 are shown in Table 4 and that at baseline and week 12 from FFQ are shown in Table 5. There were significant interactions observed between the experimental and placebo groups after the 12 week intervention for the intakes of total energy (*p* = 0.03), total fat (*p* = 0.04), saturated fat (*p* = 0.04), and carbohydrate (*p* = 0.02) based on the FFQ (Table 5). The FFQ (Table 5) also revealed a significant between group difference for carbohydrate intake (*p* = 0.04) and dietary cholesterol (*p* = 0.01). There were no significant interactions observed from the food diary analysis (Table 4), however there was a significant between group difference for protein intake (*p* = 0.02) and significant time effects were noted for total energy (*p* = 0.03), carbohydrate (*p* = 0.04), total fat (*p* = 0.02) and saturated fat (*p* = 0.01) (Table 4). The IPAQ showed that there were no significant differences in the amount of reported physical activity participation in either group at the baseline or after the 12-week intervention.

**Table 4:** Dietary intake of energy and nutrients obtained from participants’ food diary records at week 1, 4, 8 and 12 (*n* = 15 in each group).

Nutrient	Study Group	Week 1	Week 4	Week 8	Week 12	*p* Value	
Energy	Experimental	8585 ± 2705	7717 ± 1784	6742 ± 1427	6726 ± 1306	Group	0.08
(kJ/day)	Placebo	9161 ± 3316	7923 ± 1235	8660 ± 2172	8611 ± 1888 *	Time	0.03
						Interaction	0.1
Protein	Experimental	94 ± 31	97 ± 19	70 ± 12	77 ± 10	Group	0.02
(g/day)	Placebo	105 ± 47	110 ± 40	107 ± 28	110 ± 37	Time	0.1
						Interaction	0.1
Carbohydrates	Experimental	225 ± 62	200 ± 57	191 ± 53	179 ± 36	Group	0.5
(g/day)	Placebo	238 ± 83	190 ± 41	220 ± 71	200 ± 70 *	Time	0.04
						Interaction	0.4
Total fat	Experimental	79 ± 43	66 ± 24	53 ± 13 *	54 ± 14	Group	0.1
(g/day)	Placebo	84 ± 39	71 ± 22	71 ± 31 *	81 ± 21 *	Time	0.02
						Interaction	0.2
Saturated fat	Experimental	32 ± 18	23 ± 9 *	20 ± 6 *	23 ± 9	Group	0.8
(g/day)	Placebo	32 ± 17	24 ± 7 *	23 ± 12 *	23 ± 7 *	Time	0.01
						Interaction	0.2
Sodium	Experimental	2703 ± 824	2518 ± 716	2571 ± 531	2628 ± 820	Group	0.8
(mg/day)	Placebo	3072 ± 1272	2350 ± 532	2504 ± 877	2698 ± 624	Time	0.06
						Interaction	0.4
Alcohol	Experimental	7 ± 19	0.3 ± 1	0.7 ± 2	1 ± 2	Group	0.8
(g/day)	Placebo	7 ± 9	2 ± 4	2 ± 4	0.7 ± 2	Time	0.07
						Interaction	0.8

Values are expressed as mean ± SD. * *p* < 0.05, compared to baseline for each intervention.

**Table 5:** Dietary intake of energy and nutrients reported in FFQ at baseline and week 12.

Parameter	Baseline	Week 12	*p* Value	
*Energy (kJ/day)*			Group	0.06
Experimental	9012 ± 2944	6602 ± 1834	Time	1
Placebo	9659 ± 2630	8011 ± 3339	Interaction	0.03
*Protein (g/day)*			Group	0.06
Experimental	108 ± 32	85 ± 25	Time	0.8
Placebo	118 ± 40	106 ± 25	Interaction	0.3
*Total fat (g/day)*			Group	0.1
Experimental	92 ± 35	62 ± 27	Time	0.6
Placebo	97 ± 32	83 ± 37	Interaction	0.04
*Saturated fat (g/day)*			Group	0.1
Experimental	36 ± 15	24 ± 10	Time	0.9
Placebo	39 ± 15	31 ± 17	Interaction	0.04
*Polyunsaturated fat (g/day)*			Group	0.1
Experimental	14 ± 6	10 ± 5	Time	0.6
Placebo	15 ± 6	13 ± 5	Interaction	0.07
*Monounsaturated fat (g/day)*			Group	0.1
Experimental	34 ± 13	23 ± 11	Time	0.5
Placebo	35 ± 11	32 ± 14	Interaction	0.08
*Cholesterol (mg/day)*			Group	0.01
Experimental	339 ± 115	263 ± 83	Time	0.07
Placebo	408 ± 147	388 ± 177	Interaction	0.9
*Carbohydrates (g/day)*			Group	0.04
Experimental	204 ± 77	146 ± 29	Time	0.7
Placebo	223 ± 67	176 ± 45	Interaction	0.02
*Fiber (g/day)*			Group	0.2
Experimental	19 ± 6	18 ± 4	Time	0.4
Placebo	24 ± 8	21 ± 7	Interaction	0.2
*Alcohol (g/day)*			Group	0.9
Experimental	12 ± 19	7 ± 12	Time	0.8
Placebo	11 ± 11	6 ± 9	Interaction	0.4

Values are expressed as mean ± SD.

### 3.4. Appetite Assessment

There were no significant differences observed in the SQ or the amount of energy consumed from the test breakfast using the VAS in either group at the baseline or at week 12. Furthermore, significant differences were not observed in any of the measured appetite sensations after the 12-week intervention. However, both groups experienced a non-significant decrease in their satiety rating at week 12.

### 3.5. Safety and Adverse Events

In general, the *C. forskohlii* extract and placebo capsule preparations in our study were well tolerated. There were few adverse events and these were considered mild. Two participants in the experimental group experienced minor side effects in the first three weeks of the intervention period (increased bowel motions *n* =1, loose stools *n* =1). These symptoms subsided by the fourth week of the intervention.

## 4. Discussion

In the present study, supplementation with *C. forskohlii* extract in conjunction with a hypocaloric diet did not significantly affect weight loss or other anthropometric measures when compared to the placebo group. However, a significant time effect was observed in the reduction of waist and hip circumferences in both groups after the 12 week intervention (Table 2). Therefore, it appears likely that the reductions in waist and hip circumference occurred due to the dietary advice to follow a hypocaloric diet. Previous human studies have documented inconsistent results regarding changes in anthropometry with *C. forskohlii* supplementation. For instance, Henderson and colleagues [25] reported no significant changes to body weight or anthropometry, whereas Godard, Johnson and Richmond [22] recorded significant reductions in fat mass parallel to significant increases in bone mass (as determined by dual energy X ray absorptiometry (DXA) but not in total body weight in obese men after 12 weeks of supplementation with *C. forskohlii* extract. It is also important to note that the changes in waist and hip circumference occurred independent of body weight. A possible explanation for this could be that participants experienced simultaneous gains in bone and muscle mass parallel to increased fat loss as in the study by Godard, Johnson and Richmond [22]. However, the present study is limited as body composition was not measured. Methods such as DXA or 3D whole body laser scans would be useful in future studies to validate this hypothesis. Abdominal obesity is a key feature of metabolic syndrome as visceral fat is responsible for the secretion of various adipocytokines such as leptin, interleukin-6 and adiponectin. The secretion of these adipocytokines is altered in metabolic syndrome and contributes to the chronic inflammatory pathogenesis of the syndrome [37]. Changes between 1.8 and 4.1 cm in waist circumference are reported to be clinically relevant in individuals with waist circumferences ranging from 60 to 135 cm [38]. Since waist circumference is a useful indicator of visceral fat, the significant reduction in waist circumference in both experimental and placebo groups observed in our study is of potential clinical importance [1,39]. Albeit the change in waist circumference was achieved over a 12-week period and it is unknown whether the change would be maintainable over a longer period of time, thus limiting its clinical relevance.

Underreporting of food intake is a common issue in clinical practice and obesity research [40]. It was therefore deemed useful to use two different methods, that is, FFQ and self-reported food diaries to ascertain food intake throughout the study. The main findings from the present study was the significant reduction of total energy, total fat, saturated fat, carbohydrate and dietary cholesterol intakes in the experimental group based on the FFQ which suggests that dietary intake was significantly affected by *C. forskohlii* extract supplementation(Table 5). Conversely, the food diary analyses revealed a significant time effect for total energy, total fat, saturated fat and carbohydrate intakes which indicates that dietary changes were not significantly influenced by the *C. forskohlii* intervention and were instead achieved as a result of the dietary advice to maintain a hypocaloric diet. The significant time effects observed from the food diary results support the explanation that the changes in waist and hip circumference were achieved by following hypocaloric diets. The food diary results of the present study are consistent with that of Henderson, *et al.* [25] who also reported a significant time effect in dietary intake. The inconsistencies between the findings of the food diary records and FFQ are likely to be attributed to the differences in the methods of food intake reporting used (*i.e*., the FFQ presents an extensive but not exhaustive list of food items consumed during a given period of time, whereas the food diary relies on dietary recall from the participants). We recognize that the present study is limited by inter-individual variability in food intake and the subjective nature of reporting and interpreting. Furthermore, the validity and reliability of the dietary intake results would have been strengthened by measuring a biomarker such as urinary minerals or nitrogen excretion [41]. Although it is not clear how (or if) *C. forskohlii* supplementation influences dietary fat intake, emerging studies have shown that low fat taste sensitivity is associated with overweight and obesity [42,43]. Thus, we hypothesize that by reducing fat tissue (*i.e*., reducing waist circumference) in overweight and obese subjects it could have led to improved fat taste sensitivity leading to a decreased intake of fatty foods as observed in our study.

Ghrelin is a brain-gut peptide that signals hunger and increases food intake in humans [44]. Conversely, leptin is an adipocyte derived hormone that signals satiety in the hypothalamus. Circulating plasma leptin concentration is proportional to adiposity. In overweight and obesity, leptin function is thought to be deregulated whereby individuals are desensitized to the hormone and as a result still experience a heightened desire to eat despite plentiful energy stores [45]. The present study is the first to assess fasting plasma ghrelin and leptin concentrations following supplementation with *C. forskohlii* extract in humans. Previous animal studies have reported that supplementation with *C. forskohlii* extract diminished appetite (determined by measured food intake) and reduced weight gain in rodent models of obesity [46,47]. Despite the significant reductions observed in the experimental group for energy and macronutrient intakes, there were no significant changes in fasting plasma ghrelin or leptin concentrations in either group (Table 3). These findings are consistent with the appetite and satiety results obtained from the VAS. These results are also in agreement with that of Henderson, *et al.* [25] who also reported no significant results from a similar VAS method. Yet the present study is limited as only single blood samples were collected on each testing day, prior to the ingestion of the test breakfast meal. To conclusively demonstrate the effect of *C. forskohlii* extract on appetite it would be useful to take several blood samples after the ingestion of a test meal to monitor postprandial changes in ghrelin and to also correlate this data with the responses from the VAS. Furthermore, the VAS is limited in the sense that it is not completed under normal living conditions and therefore participants may experience altered appetite sensations compared to what they would in a free living context [48]. Given that the FFQ results showed that energy intake was significantly reduced in the experimental group in the present study, analysis of other peptides which regulate energy balance such as orexin, neuropeptide Y, glucagon-like-peptide-1, and peptide YY would be useful to further investigate the effects of *C. forskohlii* supplementation on appetite in humans [49].

Insulin is secreted from the pancreatic beta cells in response to rises in blood glucose concentration and promotes the cellular uptake of glucose. In the present study we found a significant decrease in fasting plasma insulin concentration and insulin resistance in the experimental group. This could be related to the significant decreases in saturated fat and carbohydrate intake in the experimental group (Table 5). Diets rich in saturated fats have been shown to influence circulating insulin concentration and insulin sensitivity [10]. Furthermore, the reduced plasma insulin concentration may be associated with a decreased abdominal fat mass as indicated by the decline in waist circumference in the experimental group. Insulin is primarily secreted when the body is in a fed state and has an overall anabolic effect on metabolism, thus causing the body to store energy [8]. In the pathogenesis of diseases such as T2DM and metabolic syndrome, there is an increase in hepatic glucose production which results in hyperglycemia and an increased secretion of insulin leading to hyperinsulinemia. Hyperinsulinemia causes tissues to become desensitized to insulin and subsequently insulin resistance develops causing chronic hyperglycemia which can have deleterious effects on the circulatory and renal systems if left untreated [6]. Thus, improving insulin sensitivity in individuals with, or at risk of developing metabolic syndrome, has an important clinical implication. In the present study, the significant change in insulin concentration and insulin resistance was independent of fasting glucose concentration (Table 3). However, the results for fasting plasma glucose concentration obtained at both the baseline and at week 12 of the study indicate that both groups were within normal levels (<5.6 mmol/L) regardless of insulin concentration [11]. This implies that insulin sensitivity could have improved after 12 weeks of supplementation in the experimental group as a lower level of insulin was required to maintain euglycemia. In comparison, Henderson and colleagues [25], reported no significant changes in fasting insulin or glucose concentrations for both the placebo and experimental group after 12 weeks supplementation with *C. forskohlii* extract.

Low HDL-C (<1.03 mmol/L for females and <1.29 mmol/L for males) is a core feature of the dyslipidaemia which occurs in the metabolic syndrome [2]. At the baseline of the study, both the experimental and placebo groups presented with relatively low levels of plasma HDL-C (Table 3). After the 12 week intervention, both groups showed a significant increase in plasma concentration of HDL-C (Table 3). The increase in HDL-C concentration may be linked to the reduction of dietary carbohydrate intake. Previous studies have reported that carbohydrate intake shows an inverse relationship with circulating HDL-C [50,51,52]. Furthermore, raised insulin and low HDL-C were found to be associated with an increased activity of hepatic lipase [53]. It has been shown that fasting insulin concentration is correlated with the hepatic lipase to lipoprotein lipase ratio, which in turn is correlated with fasting very-low density lipoprotein cholesterol [53]. The significant increase in HDL-C concentration is of potential clinical importance as HDL-C has cardio-protective and anti-inflammatory properties through its role in blocking LDL-C oxidization and thus protects against atherosclerosis and coronary heart disease [54,55]. Previous studies have reported that sustained alterations in dietary cholesterol intake have been associated with modest changes to circulating levels of TC and LDL-C in individuals with dyslipidaemia. Despite the significant reduction in dietary cholesterol intake observed in the experimental group, there were no significant changes observed in the plasma concentrations of LDL-C, TC or TG (Table 3). This might be due to the relatively short duration of intervention. Further studies of a longer duration and with a larger sample size would be useful to verify the effect of *C. forskohlii* extract on blood lipids. Moreover, circulating lipid levels are more proportionate to the endogenous cholesterol synthesized in the liver rather than to dietary cholesterol. In metabolic syndrome, the liver synthesizes increased amounts of very-low density lipoprotein cholesterol and LDL-C and decreased amounts of HDL-C [56]. Thus, data on hepatic enzyme activity related to cholesterol metabolism in animals supplemented with *C. forskohlii* would also be helpful to elucidate the mechanism of *C. forskohlii* on plasma cholesterol.

Generally *C. forskohlii* extract supplementation is very well tolerated and is recognized as safe to use [57]. However there were two reports of adverse events within the experimental group of the present study which were both related to the gastrointestinal system. This is the first controlled study to report side effects associated with the supplementation of *C. forskohlii* extract. It is plausible that the occurrence of increased bowel motions and loose stools was due to an initial increase in gastric acid secretion [58] following supplementation with *C. forskohlii* extract as forskolin has previously been shown to increase acid formation [59]. The reported gastrointestinal side effects were mild in nature and did not lead to discontinuation of the intervention. Furthermore, the symptoms subsided within four weeks of use.

Although the total number of participants satisfied the minimum number required by power analysis, the sample size was still relatively small and it is unlikely that it was a true representation of the general overweight and obese adult population, thus limiting the applicability of the results. For instance, 80% of the participants who completed the study were female. It would be useful for future studies to restrict participation to a more specific sample population and/or recruit a larger sample size. Future research directions may focus on measuring specific biomarkers to validate the reductions in energy intake and implementing DXA scans to evaluate changes in body composition. Longer term studies may also be warranted in the future to determine whether the favorable changes are maintainable for an extended period.

## 5. Conclusions

The major finding of the present study was that supplementation with *C. forskohlii* extract in conjunction with a hypocaloric diet significantly improved insulin and insulin resistance and thus may be useful in the management of metabolic risk factors. Significant increases in HDL-C were observed in both groups after the 12-week intervention. The intakes of total energy, fat, saturated fat, carbohydrate and dietary cholesterol were significantly reduced in the experimental group and thus the present study indicates that supplementation with *C. forskohlii* extract in conjunction with dietary advice may be useful for reducing dietary intake. However, further investigation is required as the dietary results obtained through different methods were inconsistent. Finally, supplementation with *C. forskohlii* extract had limited effects on anthropometric measures despite the marked reductions in dietary intake.
